# Psychometric Properties of the Obsessive-Compulsive Inventory-Child Version (OCI-CV) in Chilean Children and Adolescents

**DOI:** 10.1371/journal.pone.0136842

**Published:** 2015-08-28

**Authors:** Agustín E. Martínez-González, Tíscar Rodríguez-Jiménez, José A. Piqueras, Pablo Vera-Villarroel, Antonio Godoy

**Affiliations:** 1 Department of Developmental Psychology and Didactics, University of Alicante, Alicante, Spain; 2 Department of Health Psychology, Miguel Hernández University, Elche, Spain; 3 School of Psychology, University of Santiago de Chile, Santiago de Chile, Chile; 4 Department of Personality, Assessment and Psychological Treatment, University of Málaga, Málaga, Spain; Univ of Toledo, UNITED STATES

## Abstract

In recent years, there has been a considerable increase in the development of assessment tools for obsessive-compulsive symptomatology in children and adolescents. The Obsessive Compulsive Inventory-Child Version (OCI-CV) is a well-established assessment self-report, with special interest for the assessment of dimensions of Obsessive Compulsive Disorder (OCD). This instrument has shown to be useful for clinical and non-clinical populations in two languages (English and European Spanish). Thus, the aim of this study was to analyze the psychometric properties of the OCI-CV in a Chilean community sample. The sample consisted of 816 children and adolescents with a mean age of 14.54 years (SD = 2.21; range = 10–18 years). Factor structure, internal consistency, test-retest reliability, convergent/divergent validity, and gender/age differences were examined. Confirmatory factor analysis showed a 6-factor structure (Doubting/Checking, Obsessing, Hoarding, Washing, Ordering, and Neutralizing) with one second-order factor. Good estimates of reliability (including internal consistency and test-retest), evidence supporting the validity, and small age and gender differences (higher levels of OCD symptomatology among older participants and women, respectively) are found. The OCI-CV is also an adequate scale for the assessment of obsessions and compulsions in a general population of Chilean children and adolescents.

## Introduction

Obsessive-Compulsive Disorder (OCD) became part of a new chapter in the *Diagnostic and Statistical Manual of Mental Disorders*, *Fifth Edition* [1], entitled Obsessive Compulsive and Related Disorders. OCD is a common disorder with frequent onset in childhood or early adolescence [2]. Prevalence rates of OCD in children and adolescents are between 0.2 and 3.6% [3]. Besides OCD as a diagnostic category, OCD symptomatology in children and adolescents is an underestimated and understudied problem, with rates between 2.7 and 19% for subclinical symptoms [3–5].

In recent years, there has been a significant increase in developing assessment tools for OCD in children and adolescents [6]. According to the classification of evidence-based assessment measures [7, 8], there are three levels of empirical support for measures: 1) well-established assessment (reliability and validity demonstrated in at least two published studies by different research teams); 2) approaching well-established assessment (reliability and validity demonstrated in at least two published studies by one research team, or published studies by two research teams offering mixed psychometric results); and 3) promising assessment (reliability and validity demonstrated in at least one published study). Regarding pediatric OCD-specific measures, and according to Iniesta-Sepúlveda, Rosa-Alcázar, Rosa-Alcázar & Storch [9], the Children’s Yale-Brown Obsessive Compulsive Scale (CY-BOCS) [10] is the gold standard in the assessment of OCD due to its excellent psychometric properties observed throughout studies, but it is a long clinician-administered instrument with an interview format not useful for community settings, and its self-report format is also long for screening purposes. The Obsessive Compulsive Inventory-Child Version (OCI-CV) [11] is an approaching well-established assessment self-report, with special interest for the assessment of the dimensionality of OCD. Other self-reports, such as the Children´s Florida Obsessive-Compulsive Inventory [12], the Child Saving Inventory [13], and the Obsessive Beliefs Questionnaire-Children’s Version [14] are promising assessments for assessing symptoms and the severity of OCD, but they do not assess the obsessive-compulsive dimensionality.

Thus, the OCI-CV is an approaching well-established collective self-report to assess common dimensions or domains of OCD symptoms both in clinical an nonclinical samples. It can be used for children and adolescents between 7 and 17 years old. It consists of 21 items scored on a 3-point Likert scale (0 = Never, 1 = Sometimes, and 2 = Always). It provides seven scores: Doubting/Checking, Obsessing, Hoarding, Washing, Ordering, Neutralizing, and a total score. The OCI-CV is based on the adult version named Obsessive-Compulsive Inventory-Revised (OCI-R) [15], previously validated for its use with adolescents between 12 and 18 years old [16, 17]. Even while the new DSM-5 now considers hoarding as a separate disorder from OCD, with both being classified within the Obsessive Compulsive and Related Disorders chapter, along this manuscript we will present data following the original structure factor of the OCI-CV, which includes among other symptom domains of OCD the hoarding symptom of OCD. This framework is consistent with the idea of obsessive-compulsive spectrum disorder, according to which hoarding could be conceptualized and classified in a number of ways: as a symptom dimension or subtype of OCD, a variant of OCD (when it occurs in the absence of other OCD symptoms), or as a discrete disorder [18].

Different studies have demonstrated that the OCI-CV shows a six correlated factor solution for clinical [11, 19] and community samples [20], whereas six first-order factors grouped into a single second-order factor emerged in another community sample [21]. Regarding estimates of reliability, it has presented moderate to high internal consistency (≥ .81 for the total score and between .43 and .89 for subscales) and strong test–retest reliability (*r* = .77-.82 for the total score and *r* = .68-.89 for subscales) for both clinical and non-clinical samples [11, 13, 19, 20, 21]. With regard to evidence supporting validity, different studies indicate significant and moderate correlations of OCI-CV with other OCD measures such as, for example, the CY-BOCS (*r* = .26-.31) [11, 19, 22]. OCI-CV subscales, and the total score, have also shown association with depression and separation anxiety symptoms [20] and a strong relationship with perfectionism (*r* = .45) [23]. Concerning discriminant validity, the OCI-CV total score showed a non-significant correlation with general psychopathology (*r* = .07) [19], whereas the Children’s Saving Inventory showed relationships significantly stronger (*r* = .69) with the Hoarding subscale of the OCI-CV than with the remaining OCI-CV factors [13]. Regarding differences in gender and age, studies have shown that there are generally some differences in OCD symptoms: females are affected at a slightly higher rate than males in childhood [1]. Studies with a non-clinical adolescent sample that used the OCI-R showed that girls achieved higher scores than boys in all subscales, particularly in washing, hoarding, and neutralizing [16]. In a similar study, Rodríguez-Jiménez et al. [21] found that girls had a higher score than boys in the total OCI-CV in obsessing, hoarding, and ordering. However, other studies did not find differences according to gender [11, 19, 20]. Regarding age differences, adolescents have a tendency to score higher than children in some OCI-CV scales in clinical samples [11, 21]. However, other studies have found that scores on OCD symptoms are higher in children between 9 and 11 years compared with adolescents [20].

In spite of the evidence regarding the validity and reliability of the OCI-CV in clinical and non-clinical samples in English and European Spanish speakers, there is a lack of psychometric studies on the OCI-CV taking into account different races, ethnicities, countries, and cultures [24] in order to contribute to disseminate evidence-based assessment procedures. Furthermore, the OCI-CV could be an important scale for early detection of OCD symptoms in Latin American children and adolescents. Finally, it is worth mentioning that some of the main researchers in OCD have found support for the continuous nature of the obsessive- compulsive symptoms [25–27], which endorses the use and validation of the OCI-CV in a non-clinical population, as in this case.

Thus, the main purpose of this study was to examine empirically whether the OCI-CV is suitable for assessing OCD symptoms in a community-based sample of Chilean children and adolescents. To accomplish this, a number of aims were considered in this instrumental study: i) examine the factor structure; ii) assess internal consistency and test-retest reliability; iii) study the convergent and discriminant validity; and iv) explore the presence of gender and age differences in the OCI-CV total score and subscales.

Therefore, we propose the following hypothesis: 1) the OCI-CV scores will be grouped into the same six-factor structure (Doubting/Checking, Obsessing, Hoarding, Washing, Ordering, Neutralizing) or six factors with one second-order factor as in previous studies; 2) the internal consistency and temporal stability estimates of the OCI-CV scores will be similar to those found in previous works; 3) the association of OCI-CV scores will be higher with OCD measures than with other related constructs (anxiety and depression); and 4) age and gender differences will be found (higher levels of OCD symptomatology among older participants and women, respectively).

## Method

### Participants

The sample consisted of 816 students from two schools in the metropolitan area of Santiago de Chile and two schools in the south of the country (Sixth and Ninth regions), of which 51.3% were male. Their mean age was 14.54 years (*SD* = 2.21; range = 10–18 years).

### Instruments

- An *ad hoc socio-demographic questionnaire* designed to collect data on age, sex, and geographic area of residence.
- *Spanish version of the Obsessive Compulsive Inventory-Child Version* (OCI-CV; Foa et al. [11]). The psychometric properties of the OCI-CV were described above.
- *Spanish version of the Children’s Florida Obsessive Compulsive Inventory* (C-FOCI; Storch et al. [12]). The C-FOCI assesses OCD symptoms in children and adolescents aged 7 to 20 years. It consists of 22 items grouped in two subscales: the Symptom Checklist and Severity Scale. It has shown adequate psychometric properties for both English [12] and Spanish-speaking children and adolescents [28]. In this study, the internal consistency of the scales was α = .75 for symptoms and α = .80 for severity.
- *Short OCD Screener* (SOCS; Uher et al. [29]). The SOCS includes 7 self-report items that assess the presence of common obsessions and compulsions. This instrument showed good internal consistency, test–retest stability, a unidimensional factor structure, and excellent sensitivity to detect OCD in English [29] and Spanish children and adolescents [30]. For this sample, the internal consistency of the SOCS was α = .65.
- *Revised Child Anxiety and Depression Scale* (RCADS; Chorpita et al. [31]). This self-report consists of 47 items that assess the following symptoms: separation anxiety disorder, social phobia, panic disorder, generalized anxiety disorder, OCD, and major depressive disorder. The scale showed good psychometric properties in Spain [32] and it has been used with Chilean samples and in other countries [33, 34]. The internal consistency was good for each subscale and the total score: panic disorder α = .85, social phobia α = .82, separation anxiety disorder α = .73, generalized anxiety disorder α = .74, OCD α = .71, major depressive disorder α = .85, and total α = .94.

### Procedure

The study used the European Spanish version of the OCI-CV by Rodríguez-Jiménez et al. [21]. The questionnaire was reviewed by a group of experts to analyze whether there were differences or difficulties in reading comprehension of text among Chilean participants. Ultimately, the only item that was changed from the European Spanish version of the OCI-CV was Item 16 (see Table 1), in which the verb *tirar* was substituted by its synonym of *botar*, more frequently used in Chile.

**Table 1:** Item number, scale name / item content, scale / item mean (M) and standard deviation (SD), item factor loading (lambda), and first-order-factor loadings (gamma). Total sample (N = 816).

Item	Scale name / item content	M	SD	Gamma/Lambda
	**Obsessing**	**2.10**	**2.00**	**.68**
01	Cuando comienzo a pensar algo malo no puedo parar	.55	.61	.75
11	Tengo malos pensamientos que me molestan	.57	.64	.79
14	Me siento mal por pensamientos malos que me vienen a la cabeza sin que yo quiera	.64	.69	.87
18	Cuando se me viene a la cabeza un pensamiento malo, necesito decir ciertas cosas una y otra vez	.34	.58	.82
	**Washing**	**2.44**	**1.60**	**.66**
02	Siento que necesito lavarme y limpiarme una y otra vez	.66	.68	.83
10	Me preocupo mucho de que las cosas estén limpias	1.00	.66	.69
21	Me lavo las manos más que otros niños/as	.78	.66	.75
	**Hoarding**	**2.14**	**1.61**	**.62**
03	Acumulo tantas cosas que terminan por estorbarme	.69	.69	.84
07	Acumulo cosas que realmente no necesito	.61	.64	.80
16	No boto las cosas porque temo que podría necesitarlas más adelante	.84	.67	.73
	**Doubting/Checking**	**3.80**	**2.18**	**.93**
04	Compruebo muchas cosas una y otra vez	.87	.65	.63
05	Después de haber hecho algo, no estoy seguro de haberlo hecho realmente	.79	.62	.56
13	Incluso después de haber terminado algo, me preocupa no haberlo acabado	.84	.66	.64
15	Compruebo puertas, ventanas y cajones una y otra vez.	.56	.69	.62
20	Incluso cuando hago algo con mucho cuidado, no creo que lo he hecho bien	.74	.66	.69
	**Neutralizing**	**.91**	**1.09**	**.81**
06	Necesito contar mientras hago algo	.21	.46	.63
09	Me retraso en mis deberes escolares porque repito las cosas una y otra vez	.51	.59	.72
12	Tengo que repetir algunos números una y otra vez	.19	.47	.70
	**Ordering**	**2.69**	**1.63**	**.79**
08	Me siento mal si mis cosas no están en el orden correcto	.1.03	.71	.66
17	Me molesta que la gente cambie la forma en que yo arreglo las cosas	.89	.74	.68
19	Necesito que las cosas estén de una cierta manera	.77	.65	.80

Three weeks after the first application, the OCI-CV was re-administered to a random sample of 188 students. Nevertheless, the retest sub-sample was not equivalent to the sub-sample without retest in the following five variables: sex (more girls than boys {55% versus 45%} with retest than without retest {47% versus 53%}; Fisher’s exact statistic = .04); age (students with retest were older, mean age: 14.79 versus 14.46 –*t*(814) = -2.04; *p* = .04); generalized anxiety symptoms; RCADS-GAD (students with retest scored higher, means: 8.43 versus 7.79, *t*(814) = -2.14; *p* = .03); and depression symptoms, RCADS-MDD (students with retest scored higher, means: 9.70 versus 8.54, *t*(814) = -2.69; *p* = .01). The differences in the remaining variables assessed in Time 1 (test) were statistically non-significant.

### Ethics statement

The consent process for this study followed the same procedure that had received ethics approval for similar research implemented in secondary schools in Spain [21]. First, eligible schools were provided with information about the study, and interested schools signed written confirmation that their school wanted to participate. Second, schools provided a parental consent letter explaining the minimal risk and potential benefits associated with participation in this study and advised parents that they could withdraw their child from the study at any time. Third, all eligible children and adolescents were provided with information about the study, and they signed a written consent form to participate. The entire consent procedure and the study were approved by the ethics committee of the participating entities from Spain in this work.

The tests were applied by experienced psychologists who gave instructions and provided individual assistance to students who needed it.

### Data analysis

Due to the ample sample size, all missing values were taken out list-wise. Missing values differed neither between sexes nor between ages.

First, the IBM SPSS Statistics 22 package was used to obtain item distribution and the frequency of SOCS. Then, following the results by Foa et al. [11], two models were tested by confirmatory factor analysis by means of LISREL V. 8.8: a six correlated factor solution and a six first-order factors solution grouped under a single higher, second-order factor. As alternative hypotheses, we tested whether the OCI-CV scores are independent among them, or if all of them are grouped into a single factor. In all cases, the robust diagonally weighted least squares method was used, calculated on the polychoric correlation and asymptotic covariance matrices (LISREL, DWLS procedure). Lastly, goodness of fit measures were the following indices [35, 36]: CFI (Comparative Fit Index) and NNFI (or TLI, Tucker-Lewis Fit Index) and GFI (Goodness of Fit Index) greater than .90, and RMSEA (Root Mean Square Error of Approximation) equal to or less than .08, in addition to the chi-square corrected for non-normality. Differences between chi-squares (Δχ^2^) and CFI (Comparative Fit Index) were used to compare the goodness of fit between models [37].

Following suggestions from some authors [38], several reliability indices were calculated (using the program Factor 9.3 [39]) to estimate the reliability of the OCI-CV scores: Cronbach’s alpha, McDonald’s omega [40], and the greatest lower bound to reliability index [41]. We also examined test-retest reliability.

Successively, a MANOVA was calculated to explore the existence of possible age and gender differences in the OCI-CV scores. For it age was grouped into two categories: children/preadolescents (10–13) and adolescents (14–18) years old.

Finally, as an estimation of the evidence of convergent validity, we calculated Pearson correlations between the OCI-CV total score and other OCD measures (C-FOCI, SOCS, and OCD subscale of RCADS-30); and, as an estimation of the discriminant validity, Pearson correlations between the OCI-CV total score and measures of anxiety (RCADS-30) and depression (MD subscale of RCADS-30). Cohen’s criteria were used to assess the effect sizes of the correlations: small ≤ .20 and large ≥ .50 [42]. To test that the correlations of the OCI-CV total score with the convergent measures were higher than its correlations with the discriminant measures, we used the Meng, Rosenthal, and Rubin equations [43] (calculated with the COCOR package of the R program [44]). We concluded that correlations with convergent measures were higher than correlations with discriminant measures if the difference between both correlations was positive (e.g., correlations of convergent measures were higher than correlations of discriminant measures), the *z* statistic was significant, and the 95% confidence interval of the difference was greater than zero.

## Results

### Item analysis

The results show that all response options are chosen in all items. The mean for the item response is 0.33 points and the standard deviations range between 0.46 and 0.74, showing adequate variability (Table 1). All corrected item-total correlations exceeded the value of .30, and the removal of any item did not improve the overall alpha of the scale. Overall, the average response for the items was Never (45%), followed by Sometimes (43%), and Always (12%). However, for items 6 and 12, some 84% of the participants answered Never.

### Confirmatory factor analysis

As can be seen in Table 2, goodness of fit indices indicate that both the correlated six-factor model and the six first-order factors grouped into one second-order factor fit the data acceptably. CFI, NNFI (TLI), and GFI are equal to or greater than .90, and RMSEA is less than .08. There is no statistical difference between the model of six correlated factors and the model of six first-order factors grouped under one higher, second-order factor (Δχ^2^ = 43.89, Δ*df* = 9; *p* = .98; and both models have the same CFI value of .92). The model of a single factor and the model of six independent factors did not receive empirical support: in both cases, RMSEAs are equal to or greater than .08, and CFIs and TLI are less than .90.

**Table 2:** Results of confirmatory factor analysis of the Obsessive Compulsive Inventory-Child Version (OCI-CV).

MODELS	χ^2^ corrected for non-normality	*df*	RMSEA	CFI	NNFI (TLI)	GFI
M1. A single factor	1220.62	189	.08	.83	.81	.91
M2. Six independent factors	2075.18	189	.11	.68	.65	.63
M3. Six correlated factors	644.68	174	.06	.92	.91	.98
M4. Six first-order factors grouped under one second-order factor	688.57	183	.06	.92	.90	.97

Table 1 (last column) shows the degree of relationship (standardized lambda weights) for each item with its corresponding first-order factor, as well as the degree of relationship for each first-order factor with the higher, second-order factor (standardized gamma weights). All item weights on the factor they belong to and all the weights of first-order factors on the second-order factor are above .60.

### Reliability

The reliability (Cronbach’s alpha, McDonald's omega, and the GLB) of the OCI-CV scale is shown in Table 3. As can be observed, reliability indices of the total score and subscales are high. Although the neutralizing scale obtains a lower score than the remaining subscales, it still has a score greater than .70 (Table 3).

**Table 3:** Reliability (Cronbach’s Alpha, McDonald’s Omega, and GLB) for scales of the OCI-CV. Total sample: N = 816.

Scales	Alpha	Omega	GLB
Doubting/Checking	.76	.76	.79
Obsessing	.88	.88	.90
Hoarding	.80	.82	.82
Washing	.80	.80	.80
Ordering	.76	.76	.76
Neutralizing	.71	.73	.73
Total Score	.91	.91	.96

GLB = Greatest Lower Bound to reliability.

### Test-retest reliability

Correlations were statistically significant (*p* < .01) for all scales. The total OCI-CV presents a large 3-week test-retest correlation (*r* = .78). However, the coefficients were somewhat lower for the OCI-CV subscales (Doubting/Checking: *r* = .69; Obsessing: *r* = .70; Hoarding: *r* = .59; Washing: *r* = .68; Ordering: *r* = .63; Neutralizing: *r* = .55).

### Convergent and discriminant validity

As is shown in Table 4, correlations of the OCI-CV total score with the convergent measures (e.g., CFOCI-Symptoms, CFOCI-Severity, SOCS, and OCD scale of RCADS) were higher, ranging from .60 to .69, than their correlations with the discriminant measures (e.g., all RCADS scales, except the obsessive-compulsive scale, RCADS-OCD), which ranged from .39 to .64. In most cases, the correlation of the OCI-CV total score with the convergent measures was statistically higher that its correlations with the discriminant measures, partially supporting the convergent/discriminant validity of the OCI-CV total score. Applying the Meng, Rosenthal, and Rubin [43] criteria, the only exceptions were the following five (out of 23): correlations of the OCI-CV total score with CFOCI-Symptoms, CFOCI-Severity, and the RCADS-OCD did not differ statistically from its correlation with the RCADS total score; and correlations of the OCI-CV total score with CFOCI-severity and the RCADS-OCD did not differ from its correlation with RCADS-Panic.

**Table 4:** Convergent/Discriminant validity (N = 816).

	OCI-CV						
	Doubting/ Checking	Obsessing	Hoarding	Washing	Ordering	Neutralizing	Total
C-FOCI- Symptom Checklist	.60**	.46**	.38**	.46**	.48**	.45**	.68**
- Severity Scale	.45**	.53**	.41**	.32**	.37**	.40**	.60**
SOCS	.57**	.47**	.41**	.47**	.48**	.45**	.69**
RCADS							
- Separation Anxiety	.33**	.37**	.23**	.17**	.16**	.37**	.39**
- Social Phobia	.46**	.43**	.35**	.23**	.31**	.31**	.52**
- Generalized Anxiety	.45**	.43**	.32**	.29**	.32**	.27**	.52**
- Panic Disorder	.49**	.56**	.39**	.23**	.31**	.40**	.58**
- Obsessive-Compulsive Disorder	.55**	.55**	.36**	.31**	.37**	.37**	.62**
- Major Depression	.42**	.54**	.37**	.15**	.29**	.34**	.52**
- Total score	.57**	.61**	.43**	.28**	.37**	.43**	.66**

OCI-CV, Obsessive Compulsive Inventory-Child Version; C-FOCI, Children’s Florida Obsessive-Compulsive Inventory; SOCS, Short OCD Screener; RCADS, Revised Child Anxiety and Depression Scale.

** p < .01

### Age and gender differences

The MANOVA conducted shows significant differences related to gender (Wilks’s Lambda = .97, *F* (6, 807) = 3.68, *p* < .01), specifically in Doubt/Checking (*F* = 6.10, p < .05), Obsessing (*F* = 21.79, *p* < .001), and Hoarding (*F* = 4.59, *p* < .05), with higher means for women in all cases. All effect sizes are small (partial η^2^ = .01-.03)

The participants’ age was also significantly related to OCD symptoms (Wilks’s Lambda = .93, *F* (6, 807) = 9.46, *p* < .001). These differences are present in all factors, except for Neutralizing: Doubt/Checking (*F* = 11.43, *p* < .01), Obsessing (*F* = 14.89, *p* < .001), Hoarding (*F* = 7.85, *p* < .01), Washing (*F* = 41.56, *p* < .001), and Ordering (*F* = 27.70, *p* < .001), with older participants scoring higher. Effect sizes are small for all subscales (partial η^2^ = .01-.03), except for Washing with moderate effect size (partial η^2^ = .05). Concerning the interaction gender x age, significant differences are not found.

## Discussion

The main objective of this study was to examine empirically whether the OCI-CV is a reliable and valid instrument for the assessment of OCD symptoms in a community-based sample of Chilean children and adolescents. Overall, the OCI-CV is a suitable instrument for the assessment of the multidimensionality of OCD.

The confirmatory factor analysis of the OCI-CV in Chilean adolescents indicated an acceptable fit of data to the model of six first-order factors and one higher second-order factor, equal to that reported by Rodríguez-Jiménez et al. [21], as well as to the correlated six-factor model previously found by Foa et al. [11], Jones et al. [19], and Rosa-Alcázar et al. [20]. Thus, the scores from the Chilean sample were grouped within the same six factors as previous studies with clinical [11, 19] and non-clinical samples [20, 21], and all factors included the same items as those in the original version [11], suggesting that OCD can be conceptualized more as a spectrum of overlapping syndromes than as a single disorder.

With respect to estimates of reliability, the results specified high internal consistency for the total score (> .90) and all subscales of the OCI-CV (.71-.90) in the sample of Chilean adolescents. These results are consistent with other studies showing estimates of internal consistency greater than .85 for the OCI-CV total score and around .80 for subscales [11, 13, 19, 20, 21, 22, 45]. Furthermore, estimates of internal consistency are above the recommended value of .70 by Nunnally & Bernstein [46]. In addition, our results indicated a moderate to large 3-week test-retest reliability (*r* = .55-.78), which was similar to studies with clinical (*r* = .68-.89; Foa et al. [11]) and non-clinical samples (*r* = .70-.82; Rosa-Alcázar et al. [20]). Consequently, the stability of the OCI-CV across applications showed good psychometrics.

Regarding convergent and discriminant validity, the correlations between the total and subscale scores with other OCD instruments with empirical support (C-FOCI, SOCS, and the OCD subscale of RCADS-30) were moderate to large (*r* = .31-.69). These results are consistent with previous studies showing small to moderate correlations (*r* = .14-.52) between OCI-CV scores and different measures of OCD (e.g., CYBOCS, LOI-CV, MOCI, etc.) [11, 13, 19, 20, 21, 45]. In addition, our OCI-CV scores exhibited small to large correlations with anxiety symptoms (*r* = .16-.66) and major depression symptoms (*r* = .15-.54) measured with the RCADS-30. These findings were similar to those found in previous studies, showing a significant correlation with anxiety (*r* = .24-.62) and depression measures (*r* = .17-.47) [11, 20, 45]. Overall, these results highlight the convergent validity of the OCI-CV. On the contrary, evidence concerning discriminant validity was not sufficiently supported, since correlations with depression and anxiety measures were also significant, but lower than those with specific measures of OCD.

Concerning gender and age differences in OCD symptoms, our study highlights significant differences according to gender. Female adolescents presented significantly higher scores in the Checking, Obsessing, Hoarding, and Neutralizing subscales. Anyway, there is some controversy regarding this topic, due to the fact that some studies support these gender differences [1, 16, 21], whereas other studies do not find these differences [11, 19, 20]. Regarding age differences, adolescents show higher scores than children. These differences are consistent with studies with clinical and community-based samples [11, 21]. However, one specific study found that OCD symptoms were higher among children than among adolescents [20].

To sum up, the OCI-CV seems to be an excellent instrument for assessing OCD symptomatology in children and adolescents. The results of our study show that the OCI-CV presents psychometric properties similar to the original version when it is administered to Chilean adolescents.

However, future studies should take into account some considerations: 1) only self-report measures were used in this research, which could introduce biases such as social desirability. In this sense, future research should employ different evaluation procedures (e.g., information from parents); 2) Chilean adolescents belonging to the Mapuche ethnic group were included in this study, so future research should examine the psychometric properties of the OCI-CV according to ethnic differences (e.g., the Chilean Mapuche population); and 3) this study only included a non-clinical sample. Thus, examining the psychometric properties of the OCI-CV in clinical samples of Chilean adolescents is a pending task.

In conclusion, the OCI-CV presents different strengths, such as providing the assessment of OCD symptom severity and adequate reliability and validity supported in different populations. Overall, our data are consistent with previous literature on dimensional models of OCD and OCD symptomatology, which support the latent structure of obsessive-compulsive symptoms or dimensions [27]. Similarly, the examination of the OCI-CV in a nonclinical sample is consistent with evidence that obsessions and compulsions are universal experiences, occurring clinical and nonclinical individuals on a continuum of severity [25–27].

To sum up, following the classification of EBAs [7, 8], which are based on three levels of empirical support, the OCI-CV can be considered a well-established, or at least a promising, pediatric OCD assessment instrument. Its use as a diagnostic and screener instrument for OCD in Chilean children and adolescents, as well as an excellent measure for cross-cultural studies on OCD symptoms, can be recommended with scientific warranties.
